# Update on perioperative management of patients undergoing surgery for liver cancer

**DOI:** 10.1002/ags3.12529

**Published:** 2021-12-15

**Authors:** Masaki Kaibori, Kosuke Matsui, Mitsuo Shimada, Shoji Kubo, Kiyoshi Hasegawa

**Affiliations:** ^1^ Department of Surgery Kansai Medical University Osaka Japan; ^2^ Department of Surgery Tokushima University Tokushima Japan; ^3^ Department of Hepato‐Biliary‐Pancreatic Surgery Osaka City University Graduate School of Medicine Osaka Japan; ^4^ Hepato‐Biliary‐Pancreatic Surgery Division Department of Surgery Graduate School of Medicine The University of Tokyo Tokyo Japan

**Keywords:** liver cancer, perioperative management, review, surgery

## Abstract

Hepatocellular carcinoma is often accompanied by chronic hepatitis or cirrhosis. Preoperative evaluation of liver function and postoperative nutritional management are critical in patients with hepatocellular carcinoma who undergo liver surgery. Although the incidence of postoperative complications and death has declined in Japan over the last 10 years, postoperative complications have not been fully overcome. Therefore, surgical procedures and perioperative management must be improved. Accurate preoperative evaluations of liver function, nutrition, inflammation, and body skeletal muscle are required. Determination of the optimal surgical procedure should consider not only tumor characteristics but also the physical reserve of the patient. Nutritional management of chronic liver disorders, especially maintaining protein synthesis for postoperative protein/energy, is important. Prophylactic antibiotics are recommended for short‐term use within 24 hours after surgery. Abdominal drainage is recommended for patients with cirrhosis who may develop large amounts of ascites, who are at risk of postoperative bleeding, or who may have bile leakage due to a large resection area. Postoperative exercise therapy may improve insulin resistance in patients with chronic liver damage. Implementation of an early/enhanced recovery after surgery program is recommended to reduce biological invasive responses and achieve early independence of physical activity and nutrition intake. We review the latest information on the perioperative management of patients undergoing liver resection for hepatocellular carcinoma.

## Graphical abstract

This review summarizes a series of unique approaches to the perioperative management of patients with HCC undergoing liver resection based on the available evidence, with the goal of achieving “no mortality” and “minimal postoperative complications.” New methods for the improvement of preoperative liver function and perioperative management are likely to facilitate expansion of the indication for liver resection.

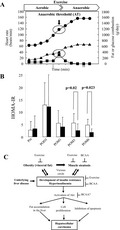

## 1 INTRODUCTION

Hepatocellular carcinoma (HCC) is the fifth most common cancer worldwide.^1^ Although most affected patients are in Asia and Africa, HCC incidence and mortality rates are increasing in North America and Europe.^2^, ^3^ In Japan, most HCCs occur in patients with chronic hepatitis and liver cirrhosis induced by hepatitis B or C virus infection. Because of advances in perioperative management, anesthesia, and operative techniques, hepatectomy for HCC has become more common.^4^ However, the postoperative mortality rate remains higher than in patients with cirrhosis or chronic hepatitis undergoing other types of surgery. The morbidity rate of patients with cirrhosis undergoing liver resection has been reported to range from 20% to 70%, with mortality rates of 5%‐21%.^5^, ^6^, ^7^, ^8^, ^9^, ^10^ Mortality rates at high‐volume centers in Japan are usually much lower, <2%,^11^, ^12^, ^13^ although morbidity rates remain relatively high. The postoperative course of these patients does not always proceed as expected, owing to various types of intraoperative stress, including blood loss and ischemia. These findings emphasize the importance of improving both surgical techniques and perioperative care in reducing the mortality and morbidity of patients with HCC undergoing liver resection. In this review, we outline the current status of and topics regarding the perioperative management of patients undergoing surgery for liver cancer based on recent evidence.

## 2 ALGORITHM FOR THE TREATMENT OF HCC AND APPROPRIATE EVALUATION OF LIVER FUNCTION BEFORE HEPATECTOMY

**FIGURE 1:**
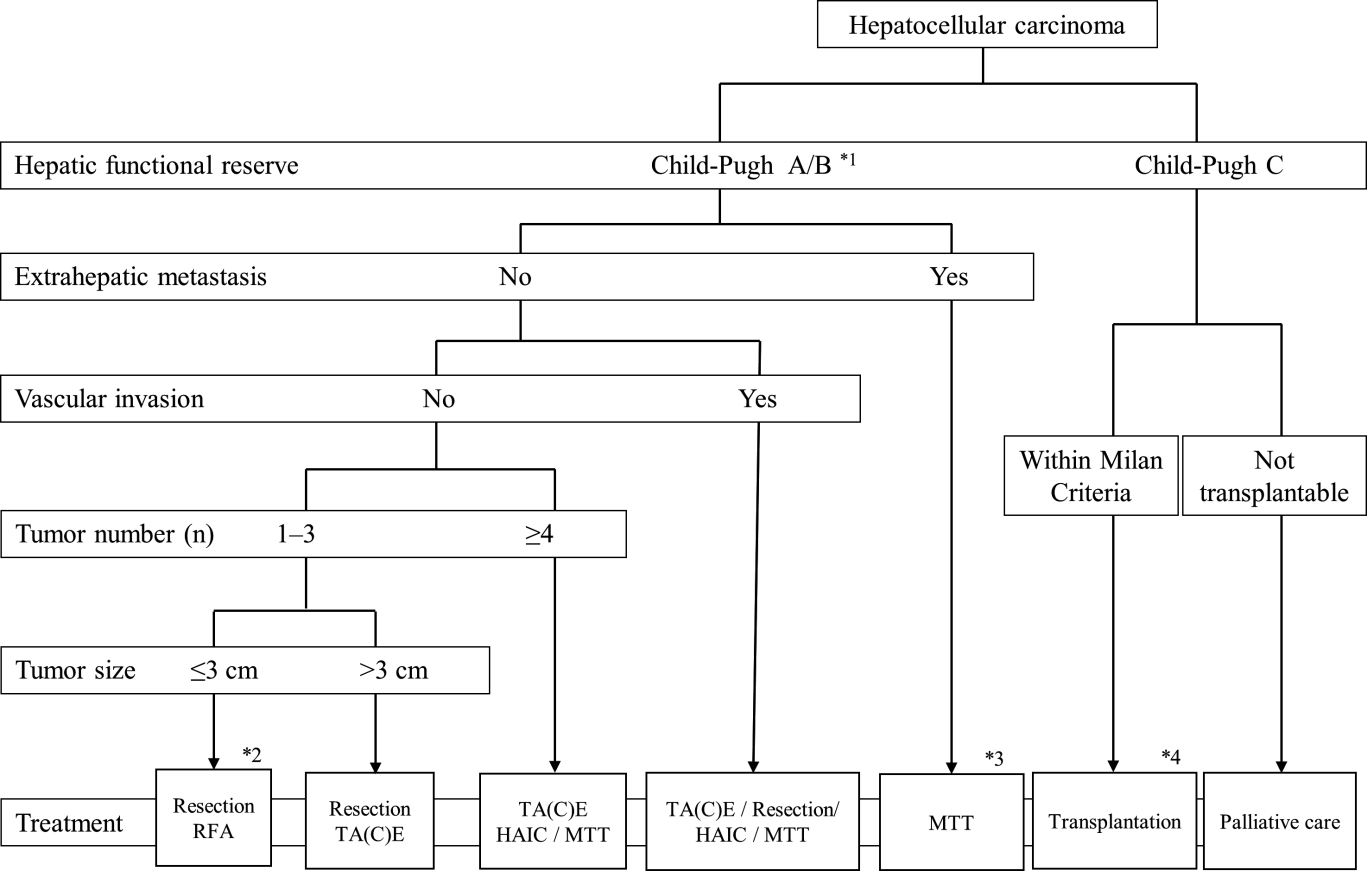
Algorithm for treatment in Japanese guidelines for hepatocellular carcinoma.^15^ This algorithm is simple and easy to memorize, consisting of five factors: (a) hepatic functional reserve; (b) extrahepatic metastasis; (c) vascular invasion, (d) tumor number; and (e) tumor size. *1: Assessment based on liver damage is recommended in the case of hepatectomy. *2: For a solitary hepatocellular carcinoma, resection is recommended as first‐line therapy, and ablation as second‐line therapy. *3: Patients with Child‐Pugh A only. *4: Patients aged ≤65 y. HAIC, hepatic arterial infusion chemotherapy; MTT, molecular‐targeted therapy; RFA, radiofrequency ablation; TA(C)E, transcatheter arterial (chemo) embolization

**FIGURE 2:**
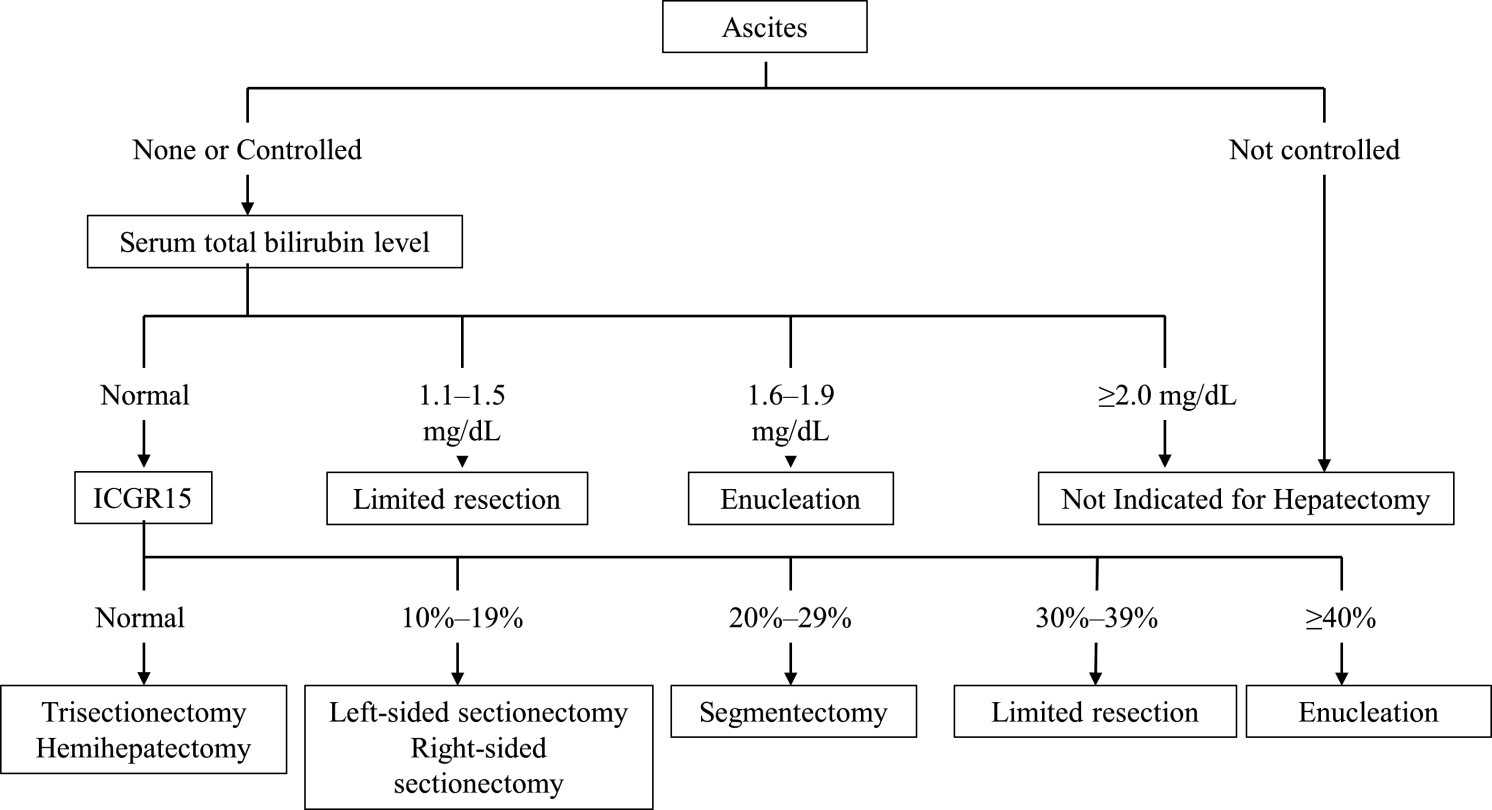
“Makuuchi's criteria” as a surgical decision tree for liver resection. Makuuchi's criteria include three factors: ascites, total serum bilirubin, and the ICGR15 (indocyanine green 15‐min retention rate). This algorithm shows the maximal area for which an operation can be performed safely (modified ref. 25)

With regard to the therapeutic strategy for HCC, the Barcelona Clinic Liver Cancer (BCLC) staging system recommended by the American Association for the Study of Liver Diseases and the European Association for the Study of the Liver is used worldwide.^14^ In Japan, the “treatment algorithm” described in the Clinical Guidelines for HCC is widely used to select the optimum treatment based on liver function and tumor status (Figure 1).^15^ The Japanese treatment algorithm differs markedly from the BCLC system with regard to HCC with concomitant portal hypertension.^16^ In the BCLC system, liver resection is not indicated if portal hypertension is present, and liver transplantation and radiofrequency ablation (RFA) are recommended. In contrast, liver resection is recommended based on the indocyanine green (ICG) retention rate at the 15‐min (ICGR15) level in the Japanese treatment algorithm, and favorable outcomes have been reported.^17^ Liver resection for HCC is chosen based on the balance between tumor status and liver function. Resection exceeding the hepatic functional reserve with the goal of cancer cure may lead to liver failure, whereas insufficient resection due to excessive safety concerns may have a high risk of early recurrence. Therefore, it is important to select the optimum surgical procedure based on the extent of the tumor and the acceptable liver resection range. As the liver reserve classification for preoperative liver function evaluation, the Child classification and its modified Child‐Pugh classification have been widely used worldwide. In particular, the presence or absence of ascites is used as an index of the degree of portal hypertension, and poor control of ascites is not indicated for surgery. In Europe and the United States, it has been common that B and C cases of the Child‐Pugh classification are not indicated for surgery, and even in cases of Child‐Pugh classification A, if portal hypertension coexists, hepatectomy is not indicated. This standard is adopted in the liver cancer treatment guidelines in Europe and the United States.^14^ In contrast, reports from Europe and the United States have stated that portal hypertension is not a contraindication for hepatectomy with more than two sections.^18^ It has been reported in Japan that reduced hepatectomy with portal hypertension is not contraindicated, because no increase in postoperative complications was observed.^17^ The ICG loading test and technetium‐99m‐garactosyl human serum albumin (^99m^Tc‐GSA) liver scintigraphy are the main quantitative preoperative evaluations of liver function for hepatectomy. Many studies of ICG load have found that it is a useful predictor of postoperative mortality.^5^, ^19^ The ICG retention rate at 15 minutes (ICGR15) has been adopted as a factor in the evaluation of liver damage by the Japan Liver Cancer Study Group^20^ and has become a standard evaluation of preoperative liver function. Yamanaka et al reported that the prediction score for the occurrence of postoperative liver failure as a surgical indication criterion, which consists of the ICGR15, amount of resection, and age, could accurately predict postoperative mortality.^21^, ^22^ Takasaki et al proposed a standard that set a different permissible amount of hepatectomy for each value of the ICG loading test.^23^ Postoperative liver failure and death within the permissible hepatectomy criteria were 2% and 0%, respectively, whereas for non‐permissible hepatectomy, they were 23% and 1%, respectively.^24^ The Makuuchi standard,^25^ which is widely used in Japan, clearly indicates whether hepatectomy is indicated and the allowable range of resection based on ascites, total serum bilirubin level, and ICGR15 (Figure 2). It has been reported that 0% of surgical deaths occurred in 1056 patients who underwent hepatectomy in compliance with this standard.^26^ Kokudo et al reported that the Albumin‐Indocyanine Green Evaluation (ALICE) grade using the serum albumin level and ICGR15 is useful for predicting the occurrence of postoperative liver failure and survival.^27^ The ALICE grade is superior to the Child‐Pugh classification in predicting outcomes after hepatectomy and may be a more useful liver function evaluation classification when combined with the presence or absence of portal hypertension.^28^, ^29^ It has been reported that ^99m^Tc‐GSA liver scintigraphy was superior to the ICGR15 in the histologic evaluation of liver damage.^30^ Evaluation of functional residual liver volume calculated from ^99m^Tc‐GSA liver scintigraphy was more informative than residual liver volume evaluation from computed tomography (CT) for predicting postoperative complications and death in patients with HCC and liver damage.^31^ However, ^99m^Tc‐GSA liver scintigraphy has facility restrictions due to the use of nuclides. In the evaluation of preoperative liver function to decide the surgical indication, in addition to information such as the Child‐Pugh classification obtained in daily clinical practice, including blood tests, many reports recommend the ICG load test as a quantitative test.^15^ For hepatectomy, it is considered appropriate to determine the indication based on the balance between the degree of liver damage estimated from the ICGR15 and the range of hepatectomy (the amount of resection required).

## 3 NUTRITIONAL STATUS OF PATIENTS WITH LIVER CANCER

### 3.1 Evaluation by biochemical and physical factors

Recent studies have proposed markers based on several nutritional or inflammation‐based prognostic indicators of HCC. Nutritional‐ or inflammation‐based markers include the prognostic nutritional index,^32^, ^33^, ^34^ controlling nutritional status (CONUT) score,^35^, ^36^, ^37^ Glasgow prognostic score,^38^ C‐reactive protein (CRP)‐to‐albumin ratio (CAR),^39^ neutrophil‐to‐lymphocyte ratio (NLR),^40^ platelet‐to‐lymphocyte ratio (PLR),^41^, ^42^ lymphocyte‐to‐monocyte ratio (LMR),^43^, ^44^ and CRP‐albumin‐lymphocyte index.^45^ Table 1 shows combined indices that can reportedly be used to estimate the nutritional or inflammatory status relevant to short‐ and long‐term outcomes after hepatectomy for HCC. While some of the algorithms are complicated, CRP and serum albumin measurements are used more often as components of combined indices.^39^

**TABLE 1:** Combined indices estimating nutritional or inflammatory status for liver cancer surgery

Index	Components (serum levels)	Results
PNI	Albumin, lymphocyte	PNI <44 was associated with higher transfusion rates and surgical outcomes,^32^ PNI <45 was the most powerful predictor of complications after hepatic resection.^33^ Patients with PNI <37 were at high risk for early recurrence and poor survival.^34^
CONUT	Albumin, lymphocyte, total cholesterol	Early postoperative CONUT score >8 was identified as a risk factor for postoperative complication III‐V.^35^ Preoperative CONUT scores >4 was predictive of worse OS and RFS.^36^ High CONUT score was an independent predictor of in‐hospital mortality after hepatectomy^37^
GPS	Albumin, C‐reactive protein	An elevated GPS was an independent prognostic indicator for OS after hepatectomy^38^
CRP/ALB ratio (CAR)	Albumin, C‐reactive protein	High CAR (>0.027) was correlated with both poor OS and DFS^39^
NLR	Neutrophil, lymphocyte	Preoperative plus postoperative ratio was a prognostic factor for OS^40^
PLR	Platelet, lymphocyte	High PLR level (≥150) is a good indicator to predict recurrence beyond the Milan criteria.^41^ High PLR indicates a higher rate of extrahepatic metastasis of HCC.^42^
LMR	Lymphocyte, monocyte	Both LMR and NLR might be preferable independent prognostic factors for DFS.^43^ Elevated preoperative LMR (≥4.01) was independently associated with poor OS and DFS^44^
CRP‐albumin‐lymphocyte index	C‐reactive protein, albumin, lymphocyte	OS and RFS were worse with an index of 5 or higher^45^

Abbreviations: CAR, C‐reactive protein (CRP) to albumin ratio; CONUT, controlling nutritional status; DFS, disease‐free survival; GPS, Glasgow prognostic score; LMR, lymphocyte to monocyte ratio; OS, overall survival; PFS, progression‐free survival; PLR, platelet to lymphocyte ratio; PNI, prognostic nutritional index.

The use of sarcopenia to predict outcomes in patients with cancer has attracted more attention, including those with HCC^46^, ^47^, ^48^ or colorectal liver metastases (CRLM)^49^, ^50^ undergoing hepatic resection. Previous studies also demonstrated that sarcopenia increased the risk of postoperative morbidity and longer hospital stay, as well as readmission rates, after partial liver resection for CRLM.^51^, ^52^ Those studies focused only on skeletal muscle mass, as assessed by CT of skeletal muscle area. In contrast, a few reports have described the deterioration of muscle quality associated with muscle fat deposition. The usefulness of intramuscular adipose tissue content in hepatectomy for HCC^53^, ^54^ and CRLM^55^ has been reported. Preoperative sarcopenia and/or intramuscular adipose tissue content might be considered a new selection criterion for hepatectomy in patients with liver cancer.

### 3.2 Nutrition therapy

Because many cases of hepatectomy for HCC coexist with chronic hepatitis and cirrhosis, these patients often have energy/substrate metabolic disorders. Specifically, it has been reported that nutritional disorders occur frequently in patients with liver cirrhosis; 84% and 95% of patients with Child‐Pugh classifications B and C, respectively, are undernourished, and 45% of patients with Child‐Pugh classification A are also undernourished.^56^ The main characteristic of nutritional disorders in patients with liver cirrhosis is malnutrition of protein and energy, and a decrease in the burning ratio of glucose as an energy‐burning source and an increase in the burning ratio of fat are observed.^57^ These nutritional disorders in patients with cirrhosis are closely related to the incidence of complications after hepatectomy, and the importance of perioperative nutrition therapy has been noted.^58^ In principle, oral and enteral nutrition should be prioritized in nutrition therapy to maintain intestinal function. The European Society for Clinical Nutrition and Metabolism (ESPEN) guidelines also recommend early oral nutrition for surgical patients to restore intestinal function, and early postoperative nutrition therapy for patients with liver cirrhosis to prevent postoperative complications.^59^ The results of some studies on branched‐chain amino acid (BCAA) administration and immunonutrition therapy (immunonutrition) in liver surgery complicated with liver cirrhosis are described as follows. Fan et al^58^ reported a randomized controlled trial (RCT) of 124 patients who underwent hepatectomy for HCC who were divided into a perioperative nutrition therapy group that received an infusion of an emulsion of dextrose, BCAA, and medium‐chain triglycerides and a non‐nutrition therapy group. The frequency of postoperative complications was significantly reduced in the nutrition therapy group, and the incidence of infectious complications was 17% and 37% (*P* = .02) in the nutrition therapy and non‐nutrition therapy groups, respectively. Furthermore, the effect of these nutrition therapies on postoperative complications was remarkable in patients with cirrhosis who underwent major hepatectomy. Shirabe et al reported an RCT of 26 patients who underwent hepatectomy in which nutritional components containing BCAAs were administered by intravenous or enteral routes. The incidence of infectious complications was 31% in the intravenous route group and 8% in the enteral route group, demonstrating the superiority of the enteral route as a nutritional route after hepatectomy.^60^ Enteral nutrition may induce retention of the intestinal mucosa, activation of the immune system in the mesenteric lymph nodes, and increased IgA production in patients with liver cirrhosis at high risk of bacterial translocation; therefore, the authors recommended enteral nutrition early after surgery. Recently, preoperative administration of BCAA in patients undergoing hepatic resection was shown to be effective for preventing ascites, plural effusion, or both, as well as for improving albumin metabolism and reducing the risk of complications and duration of hospital stay.^61^, ^62^ Previous reports have addressed the effects of BCAA on HCC recurrence after surgery^63^, ^64^; however, no definitive conclusions have been reached. Hachiya et al reported the long‐term prognosis of BCAA administration for patients with HCC who underwent hepatectomy.^65^ They demonstrated that oral BCAA supplementation could not reduce the risk of recurrence after hepatic resection in HCC; however, the results suggested that BCAA supplementation may be beneficial for selected patients who were younger and had mildly impaired glucose tolerance.

Four studies on the efficacy of probiotics and prebiotics for patients who undergo hepatic resection have been published.^66^, ^67^, ^68^, ^69^ A meta‐analysis including these studies showed that administration of probiotics and/or prebiotics prior to the day of surgery decreases the infection rate post‐liver resection and could shorten the duration of hospitalization and antibiotic use.^70^ However, as the quality of the evidence was low, further research is required. There were three reports of perioperative immunonutrition therapy with omega‐3 fatty acids.^71^, ^72^, ^73^ Two RCTs showed that intravenous administration of omega‐3 lipids for 5 days after hepatectomy improved postoperative liver function and suppressed the occurrence of complications.^72^ However, one RCT did not show these effects.^73^

## 4 PROPHYLACTIC ANTIBIOTICS

Hirokawa et al^74^ reported the results of an RCT of the administration of prophylactic antibiotics to patients scheduled to undergo liver resection. The non‐postoperative antibiotic group (n = 95) received 1.0 g of flomoxef sodium (FMOX) 30 minutes before surgery but was not given FMOX after surgery. The antibiotic group (n = 95) was given intravenous FMOX 1.0 g every 12 hours for 3 days after surgery. The groups did not differ significantly for signs of infection (21.3% vs 25.5%, *P* = .61), incidence of systemic inflammatory response syndrome (11.7% vs 17.0%, *P* = .41), infectious complications (7.5% vs 17.0%, *P* = .07), surgical site infection (SSI; 10.6% vs 13.8%, *P* = .66), and remote site infection (2.1% vs 8.5%, *P* = .10). Takayama et al^75^ reported that the incidence of SSI was 9.5% and 9.8%, respectively, in a non‐inferiority study comparing 232 patients who received 1 g of FMOX up to 6 hours after surgery (1‐day group) and 235 patients who received it for up to 2 days (3‐day group; *P* = .001 for non‐inferiority). In a retrospective study, after propensity score matching, there was no difference in the incidence of SSI and distant site infections when comparing administration of FMOX up to 24 hours after surgery and administration for 3 days after surgery in patients undergoing open and laparoscopic hepatic resection.^76^ Based on these results, it is recommended that prophylactic antibiotics be administered within 24 hours after hepatectomy.

## 5 PROPHYLACTIC ABDOMINAL DRAINAGE

Routine prophylactic abdominal drainage was unnecessary or contraindicated for patients who underwent elective hepatectomy in RCTs, because drain placement increased the frequency of drain‐related complications, wound complications, sepsis and infectious fluid retention, and significantly increased the length of hospital stay.^77^, ^78^, ^79^, ^80^ On the other hand, in patients with liver cirrhosis accompanied by portal hypertension, one report recommended drain placement, because abdominal drainage reduced complications related to postoperative ascites and shortened the length of hospital stay.^81^ It was also reported that abdominal drainage should not be placed in patients unless they were at high risk of bleeding and bile leakage.^82^ Late‐onset bile leakage, which could lead to serious complications, such as sepsis, occurred in patients without abdominal drainage.^83^ Intra‐abdominal infection should also be treated as soon as possible, because it may induce refractory bile leakage with serious complications. Some reports recommended drain placement in cases of therapeutic usefulness for bile leakage and intraperitoneal fluid retention due to drainage,^84^, ^85^ for the possibility of predicting bile leakage by monitoring the bilirubin concentration in drainage,^85^, ^86^ for patients at high risk of bile leakage, such as those with biliary tract reconstruction, exposure of major Glisson's capsule, and with bile leakage observed during surgery.^87^ It was also reported that prophylactic abdominal drainage was not essential for living‐donor liver transplant donor hepatectomy.^88^ Unlike other abdominal organ resections, hepatectomy is often associated with chronic liver damage, and it is necessary to pay attention to bleeding, bile leakage, and intractable ascites. Regarding the pros and cons of drain placement during elective hepatectomy, RCTs have been performed since the 1990s, but they had some limitations, such as a small number of patients and the methods used for evaluation. Therefore, it was necessary to consider the degree of coexisting liver damage and the surgical procedure. More careful consideration is required for abdominal drainage in living‐donor liver transplant donor surgery performed on healthy people, and it is also necessary to consider the pros and cons of drain placement in laparoscopic hepatectomy, which has been increasing in recent years. It is currently considered that the presence or absence of abdominal drainage in elective hepatectomy should be determined in consideration of the risk of bleeding and bile leakage. The Centers for Disease Control and Prevention (CDC) guidelines for prevention of SSI recommend “if a drain is needed, use a closed drain and remove it as soon as possible.”^89^ Some reports demonstrated that it is desirable to remove the drain within 2‐3 days after surgery if there is no problem with the drainage properties.^85^, ^90^, ^91^

## 6 EXERCISE THERAPY

**FIGURE 3:**
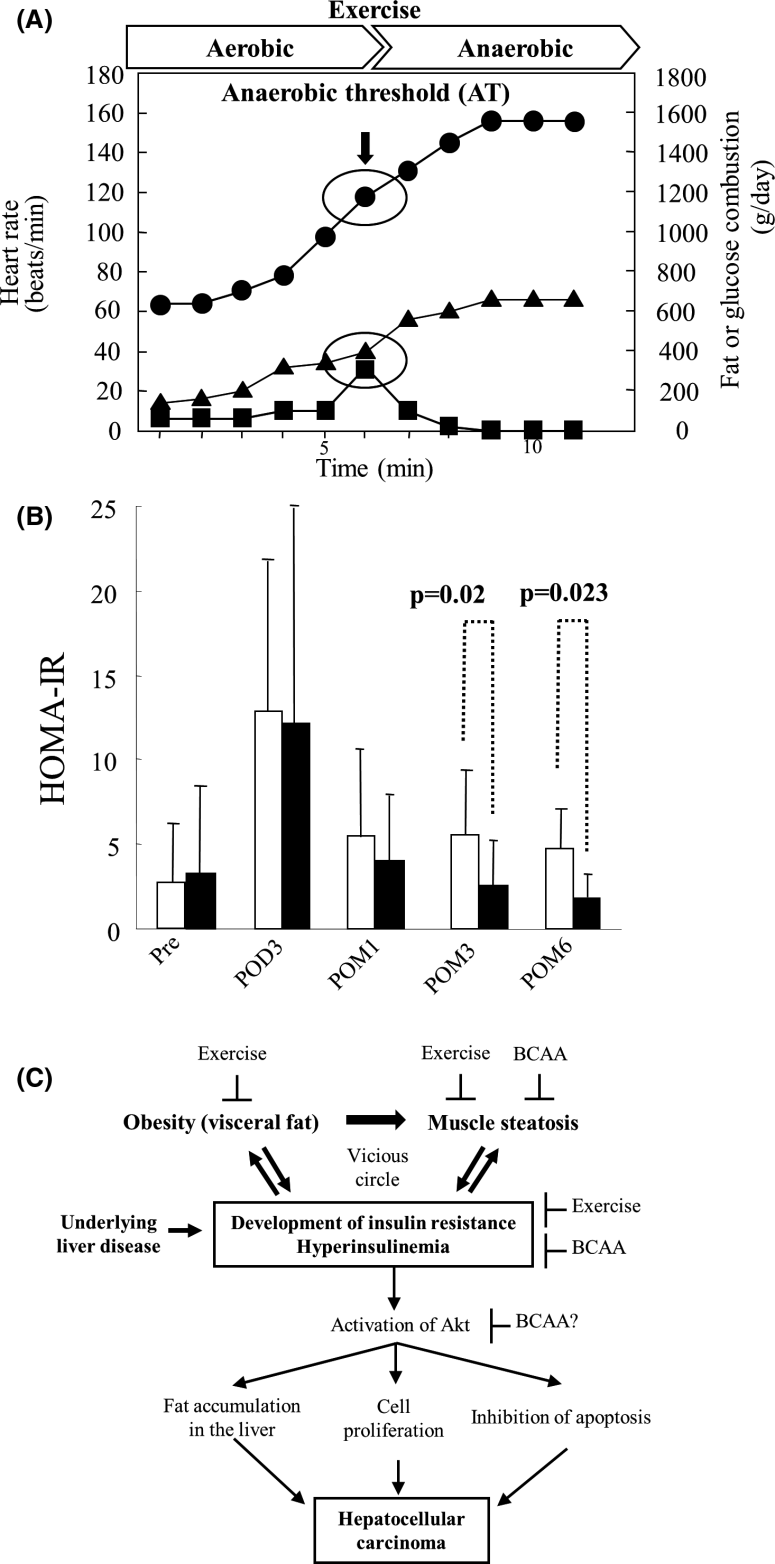
Exercise therapy. (A) Cardiopulmonary exercise test. The anaerobic threshold (AT) was set at the break point between carbon dioxide production and Vo_2_, or the point at which the ventilatory equivalent for oxygen and end‐tidal oxygen partial pressure curves reached their respective nadirs before beginning to increase again. Thus, the AT was set at a maximum point of fat combustion. ● heart rate; ▲ glucose combustion; ■ fat combustion. (B) Effect of exercise on insulin resistance in patients with HCC with hepatic impairment. HOMA‐IR in the diet group (□) and the exercise group (■). HOMA‐IR, homeostasis model for assessment of insulin resistance; POD, postoperative day; POM, postoperative month. (C) Estimating the mechanism of hepatocellular carcinoma development due to exacerbation of insulin resistance. There is a vicious cycle among obesity, muscle steatosis, and the development of insulin resistance in patients with liver diseases. Exercise and/or BCAA therapy is considered to suppress the deterioration of the condition. Akt; protein kinase B; BCAA; branched‐chain amino acid

Dynamic assessment of preoperative exercise capacity may be a useful predictor of short‐ and long‐term postoperative prognosis. Cardiopulmonary exercise (CPX) testing measures oxygen uptake at increasing levels of work and predicts cardiopulmonary performance under stress, such as after surgery. Among older patients undergoing major abdominal surgical procedures, most deaths from cardiopulmonary complications occur in those with an anaerobic threshold (AT) <11 mL/min/kg.^92^, ^93^ The AT is defined as the point during exercise at which oxygen demand outstrips oxygen delivery, and metabolism starts to become anaerobic. The AT is a measure of the ability of the cardiopulmonary system to deliver adequate oxygen to tissues, and it has the advantage of being independent of patient motivation. To date, few studies have examined the usefulness of pre‐ and postoperative CPX testing in patients undergoing hepatectomy.^94^, ^95^ Recently, Kaibori et al reported that in patients with HCC and hepatic impairment undergoing liver resection, exercise significantly decreased body mass and fat mass, as well as insulin resistance, 6 months postoperatively.^96^ Maintenance of postoperative physical strength and earlier resumption of daily activities could be possible by intensifying perioperative and postoperative exercise. An exercise program was tailored for each patient. Exercise was started as soon as possible after diagnosis, up to 1 month preoperatively, and was resumed from 1 week postoperatively and continued for 6 months. The program consisted of three 60‐minute exercise sessions per week. Each session included 5 minutes of stretching exercises, 30 minutes of walking at an intensity based on the AT of each patient, 20 minutes of targeted stretching exercises, and 5 minutes of cooling down with stretching. Patients with HCC (N = 51) were randomized to receive diet therapy alone (n = 25) or exercise in addition to diet therapy (n = 26). Whole body mass and fat mass in the exercise group compared with the diet group were significantly decreased at 6 months postoperatively. Fasting serum insulin and the homeostasis model assessment score were also significantly decreased (Figure 3). At 6 months, the AT and peak oxygen consumption were significantly increased, while serum insulin and insulin resistance significantly improved in a high frequency exercise subgroup compared with a low frequency group (Figure 2).

## 7 ENHANCED RECOVERY AFTER SURGERY

Fast‐track or enhanced recovery after surgery (ERAS) programs following surgical interventions are now within the standard of care for patients with several surgical indications.^97^, ^98^, ^99^, ^100^, ^101^ These programs use a multimodal approach to maximize effectiveness and minimize cost, thus optimizing perioperative care pathways.^102^ ERAS programs have been associated with reductions in complications, duration of hospital stay, and hospital costs in colorectal surgery.^97^ Recently, ERAS programs have also been introduced in liver surgery. Wang et al demonstrated in a meta‐analysis that the implementation of ERAS programs in liver surgery appears to be feasible and efficient and could significantly reduce overall morbidity, hospital stay and costs, intraoperative blood loss, and time to bowel function recovery without increasing rates of mortality, readmission, or transfusion.^103^ They also found that ERAS programs reduced morbidity in open and laparoscopic surgery equally, whereas hospital stays were reduced more obviously in laparoscopic surgery. However, it is unclear why the implementation of ERAS programs resulted in the reduction of intraoperative blood loss, which is largely dependent on the surgical technique. We consider that laparoscopic surgery, unlike open surgery, is effective for reduction in surgical bleeding because it requires minimal peeling due to the magnifying effect and reduces damage to tissues around the liver. The results of RCT and meta‐analysis have shown that the introduction of ERAS programs reduced the frequency of complications as well as improved early postoperative recovery.^103^, ^104^, ^105^, ^106^, ^107^ Among programs, pain management is an important factor, and Hausken et al reported that intravenous patient‐controlled analgesia (IV‐PCA) was non‐inferior to epidural anesthesia.^106^ Most of the patients in these studies underwent hepatectomy for colorectal liver metastases. Few studies have analyzed the effect of ERAS programs on patients with diseased livers who underwent hepatectomy for HCC. Kaibori et al^105^ compared clinicopathologic factors, surgical factors, and outcomes of patients who underwent extended hepatectomy (defined as resection of more than two sections) for HCC coexistent with chronic hepatitis and cirrhosis, before and after the introduction of an ERAS program. Operating time and postoperative hospital stay were significantly shorter, and total volume infused during surgery was significantly lower for the ERAS group than for the control group. Although the percentage of patients with retention of abdominal drainage was significantly smaller in the ERAS group, the frequency of abdominal paracentesis in patients without intraoperative abdominal drainage was higher. Oral dietary intake and ability to walk stably occurred significantly earlier in the ERAS group. Postoperative serum concentrations of albumin and cholinesterase were significantly higher in the ERAS group than in the control group. We concluded that a multimodal ERAS program was feasible and effective for patients with chronic liver diseases undergoing extended liver resection for HCC.

**FIGURE 4:**
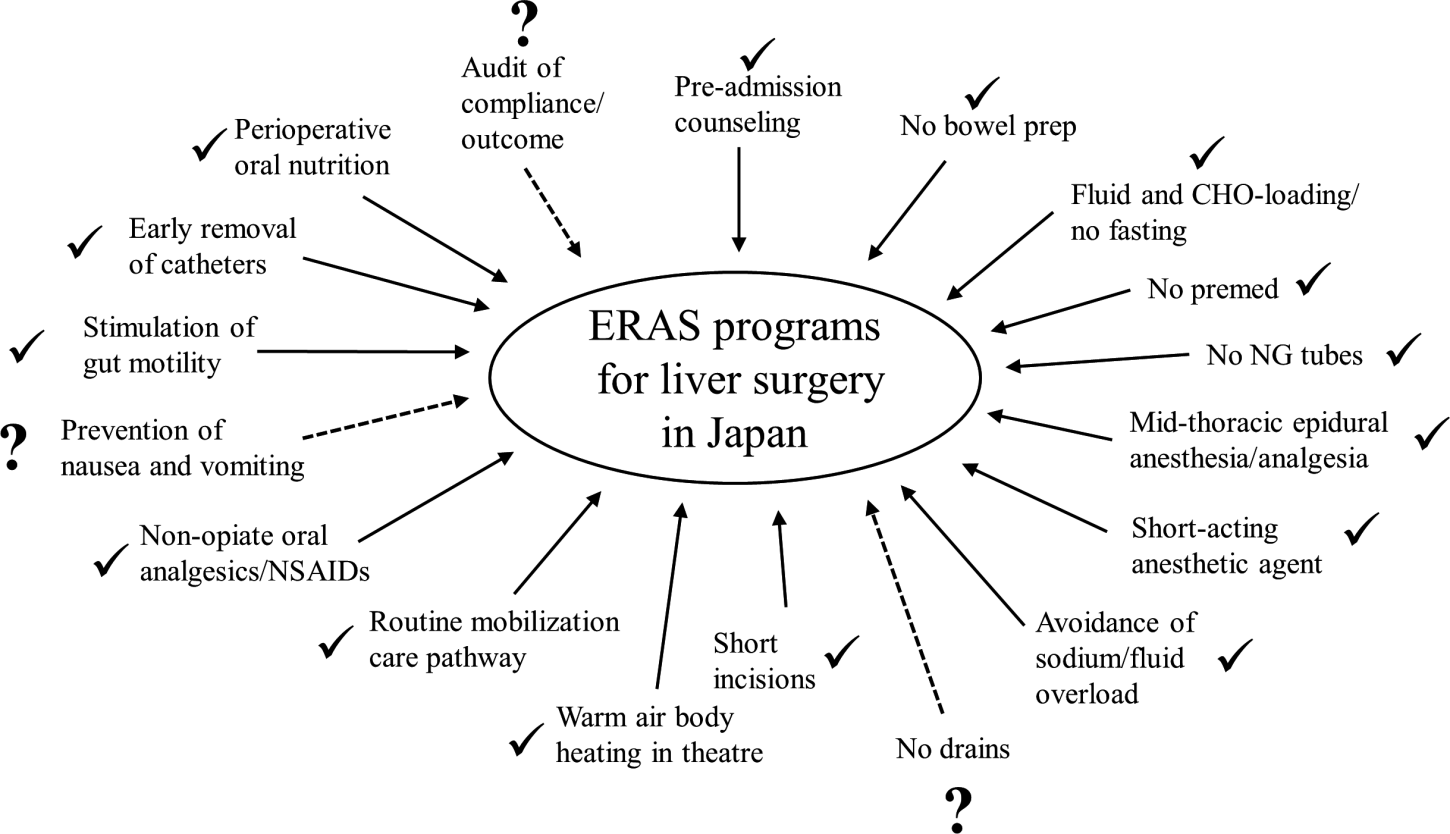
Enhanced recovery after surgery (ERAS) program items performed in patients undergoing hepatectomy in Japan. ERAS program items that have already been implemented at many facilities in Japan are marked with a check mark, and items that have not yet been implemented sufficiently are marked as question marks

We speculate that the spread of ERAS programs in hepatectomy in Japan will be as follows. There are 15 ERAS items essential for liver surgery: “Pre‐admission counseling,” “No bowel preparation,” “Fluid and CHO‐loading/no fasting,” “No pre‐anesthetic medication,” “No routine nasogastric tubes postoperatively,” “Epidural analgesia,” “Short‐acting anesthetic agent,” “Avoidance of sodium/fluid overload,” “Short incision,” “Warm air body heating in theatre,” “Early mobilization (routine mobilization care pathway),” “Non‐opiate oral analgesics/NSAIDs,” “Early feeding (stimulation of gut motility and perioperative oral nutrition),” and “Early removal of catheters.” However, “No surgical drains,” “Prevention of nausea and vomiting,” and “Audit of compliance/outcome” are not yet widespread components of ERAS programs in Japan (Figure 4).

## 8 CONCLUSIONS

This review summarizes a series of unique approaches to the perioperative management of patients with HCC undergoing liver resection based on the available evidence, with the goal of achieving “no mortality” and “minimal postoperative complications.” We believe that general perioperative management of patients undergoing surgery for liver cancer is basically the same, even when there is a wide variety of patient and surgical factors. Whether the associated liver disease is normal vs severe cirrhosis, and whether the surgery is laparoscopic vs open hepatectomy, we perform the perioperative management described above in the same way. New methods for the improvement of preoperative liver function and perioperative management are likely to facilitate expansion of the indication for liver resection.

## DISCLOSURE

Funding: All authors hereby declare that there is no potential or actual personal, financial, or political interest related to this study.

Conflict of Interest: None.

Author Contribution: MK drafted and wrote the manuscript. KM, MS, SK, and KH participated in the study design and helped draft the manuscript. All authors contributed to the interpretation of the findings, read, and approved the final manuscript.
